# Editorial: Biofluid Extracellular Vesicles and Their Involvement in Animal Reproductive Physiology

**DOI:** 10.3389/fvets.2021.747138

**Published:** 2021-09-21

**Authors:** Islam M. Saadeldin, Ahmed Gad, Pascal Mermillod

**Affiliations:** ^1^College of Veterinary Medicine, Chungnam National University, Daejeon, South Korea; ^2^Institute of Animal Physiology and Genetics, Czech Academy of Science, Libechov, Czechia; ^3^Institut National de Recherche pour l'Agriculture, l'Alimentation et l'Environnement (INRAE), Physiologie de la Reproduction et des Comportements, UMR 085 Inra-Cnrs-Université de Tours-Haras Nationaux, Nouzilly, France

**Keywords:** extracellular vesicles, exosomes, physiology, reproduction, biomarker

Recent advances have unraveled the complexity of molecular signals that allow the communication between different reproductive cell types for maintaining normal reproductive functions. This kind of communication is controlled by a myriad of factors acting through autocrine, paracrine, and endocrine signaling pathways (1). In the last decade, extracellular vesicles (EVs) have emerged as playing a significant role in regulating and facilitating this dialogue. EVs, including exosomes and microvesicles, are membrane-enveloped particles secreted by living cells to the surrounding microenvironment and body fluids, and contain proteins, messenger RNA (mRNA), and microRNAcargoes. In different mammalian species, EVs have been detected in all reproductive biofluids with emerging evidence on their roles in gametogenesis, fertilization, early embryo development, and implantation (2–6).

Recognized researchers in this field worldwide have been invited to contribute to the current Research Topic. We received three original articles and three reviews discussing the current advances in EVs isolation and characterization for a better understanding of sperm physiology, ovarian follicles function, and oviduct secretions during pregnancy in different animal models. Profiling the protein and RNA cargo in these EVs provides a powerful diagnostic tool for accurate and non-invasive assessment of male and female reproductive functions. Moreover, the EVs contents exert possible pleiotropic effects on both male and female reproductive tissues and germ cells, as well as on embryo early development, implantation, and further fetal growth. Qamar et al. reviewed the active participation of EVs with the male and female reproductive systems in the regulation of different physiological events such as oocyte maturation, fertilization, and embryo and fetal development. To illustrate the EVs functions in the ovary, Uzbekova et al. profiled the proteins within bovine follicles EVs of 53.6 ± 23.3 nm in diameter and identified 322 unique proteins, which originated from granulosa cells and from other cells. These proteins may be involved in the maintenance of follicular homeostasis and may affect oocyte developmental competence. Moreover, Mazzarella et al. investigated the micro RNAs cargo of oviduct fluid small EVs (sEVs) of around 140 nm in size in pregnant cows. They found that some unique miRNAs (bta-miR-126-5p, bta-miR-129, bta-miR-140, bta-miR-188, bta-miR-219, bta-miR-345-3p, bta-miR-4523, and bta-miR-760-3p) were up-regulated in pregnant cows at 120h after ovulation induction and one miRNA (bta-miR-331-5p) was up-regulated in non-pregnant cows. The results suggest that initial embryonic-maternal communication is mediated through the EVs in the oviduct, and the presence of gametes and the embryo can modulate miRNAs contents of oviduct EVs. Regarding the male-related factors, Xu et al. examined the RNA contents of boar seminal plasma EVs by NGS and identified many RNA classes, including mRNA (25%), PIWI interacting RNA (15%), and miRNA (9%). A total of 288 miRNAs with 37 novel miRNAs, and 19,749 piRNAs, were identified in the boar seminal plasma EVs. Particularly, ssc-miR-21-5p was identified, which may confer negative effects on boar sperm fertility based on a dual-luciferase reporter experiment.

Challenges still remain in isolating EVs from the minute embryo culture medium quantity with low EVs concentration, and much effort is required to resolve the use of these EVs as biomarkers for non-invasive determination of embryo developmental potential. For that, Talebjedi et al. reviewed the conventional methods for isolating EVs and compared them with the most advanced microfluidics methods. Moreover, they discussed the role of embryonic EVs as biomarkers of embryo quality which could be implemented in the future as a non-invasive diagnostic test for embryo selection. Then they showed the promising potentials in which microfluidics technologies could allow researchers to overcome the challenges of embryonic EVs isolation and be used as a fast and user-friendly tool. Of note, Gebremedhn et al. summarized the potential roles of EVs in mediating the follicular cells communication and shuttle the effects of environmental and metabolic stressors in various reproductive cells. Interestingly, EVs are involved in shuttling protective or rescuing molecular signals during oxidative, heat, and metabolic stress. Their future potential usage as means of targeted delivery of molecules to alleviate the effect of different stressors on oocytes was also discussed.

All together, these papers show that a better understanding of the molecular mechanisms mediating the embryonic-maternal crosstalk through EVs will lead to the development of new regulating agents (7), as well as novel theranostic tools for judging sperm, oocyte quality, and embryo developmental potential and either supporting or hindering normal reproductive functions (Figure 1).

**Figure 1:**
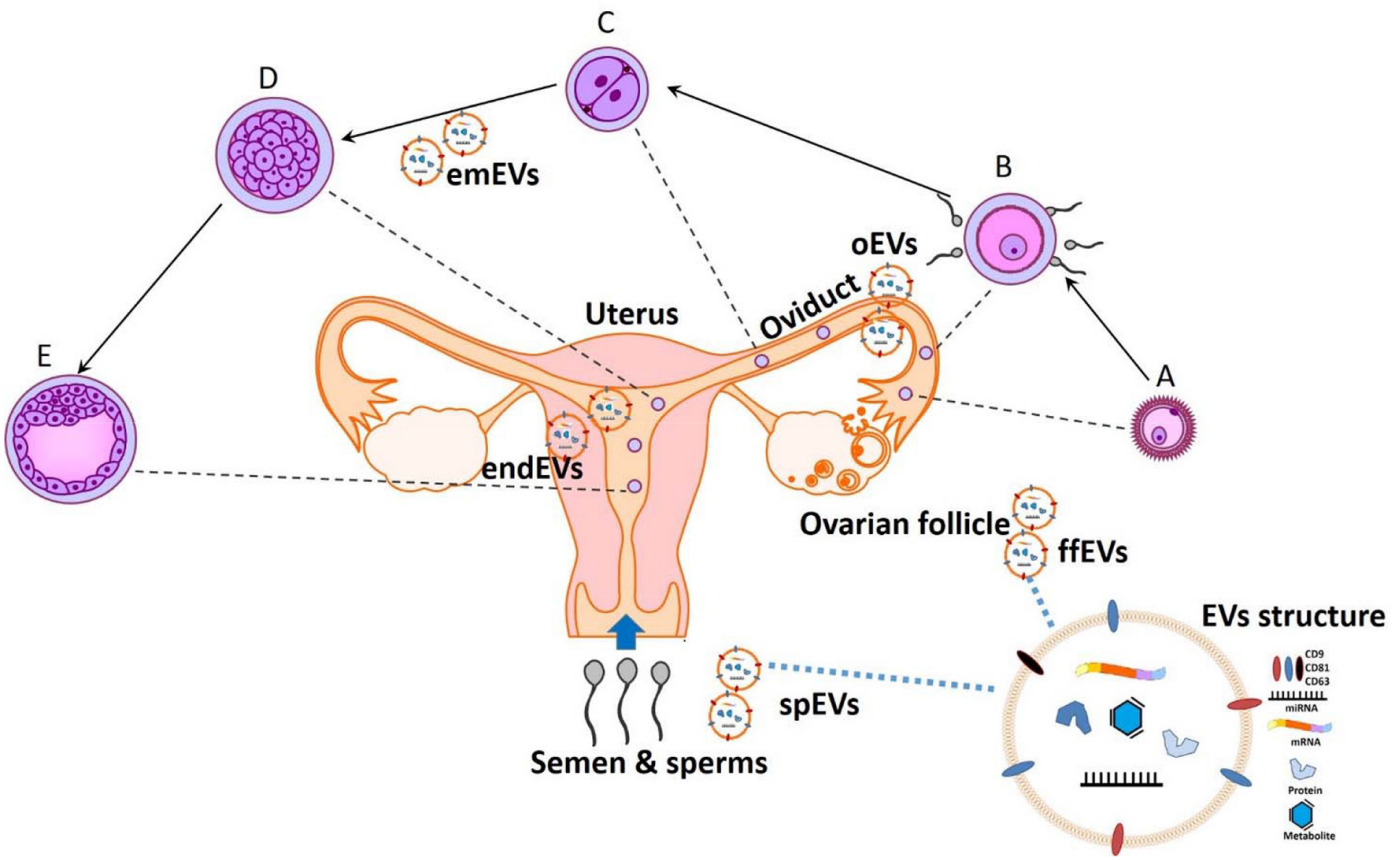
The potential theranostic approaches for embryonic-maternal microenvironments. A- Ovulated oocyte, B- Fertilization, C- Zygote cleavage, D-Morula, and E- Blastocyst formation. Extracellular vesicles (EVs) are secreted in all reproductive fluid including seminal plasma (spEVs), follicular fluid (ffEVs), oviduct (oEVs), and endometrium (endEVs), as well as from the gametes and preimplantation embryos (emEVS). Recent advances in analyzing the cargo contents of EVs will provide a paradigm for diagnostic and designing therapeutic tools for improving or opposing the reproductive physiology.

## Author Contributions

All authors listed have made a substantial, direct and intellectual contribution to the work, and approved it for publication.

## Conflict of Interest

The authors declare that the research was conducted in the absence of any commercial or financial relationships that could be construed as a potential conflict of interest.

## Publisher's Note

All claims expressed in this article are solely those of the authors and do not necessarily represent those of their affiliated organizations, or those of the publisher, the editors and the reviewers. Any product that may be evaluated in this article, or claim that may be made by its manufacturer, is not guaranteed or endorsed by the publisher.
